# Beyond diagnostic accuracy: navigating the artificial intelligence data tsunami toward value-based implementation in gastroenterology

**DOI:** 10.3389/frai.2026.1943025

**Published:** 2026-10-02

**Authors:** Konstantinos Ekmektzoglou, Theodore Rokkas

**Affiliations:** 1 Medical School, European University of Cyprus, Nicosia, Cyprus; 2 First Department of Gastroenterology, Henry Dunant Hospital Center, Athens, Greece

**Keywords:** artificial intelligence, cost-effectiveness, data tsunami, gastroenterology, human factors

## Abstract

Artificial intelligence (AI) in gastrointestinal (GI) endoscopy has evolved from its early application in simple edge detection to highly sophisticated technologies designed to improve detection of neoplastic and preneoplastic lesions and support clinical decision-making. However, this rapid technological evolution has generated a “*data tsunami*” or “*datanami*”: an overwhelming and continuously expanding volume of information that, frequently, exceeds the ability of clinicians and patients to process, interpret, and apply it effectively. Substantial investments in AI have been driven by expectations of improved efficiency and reduced healthcare costs. Nevertheless, the metrics used to justify the adoption of AI-assisted diagnostic and therapeutic strategies remain inadequately standardized, leaving the true cost-effectiveness of these technologies uncertain. Current AI research in gastroenterology has primarily focused on improving diagnostic performance rather than evaluating long-term clinical outcomes or economic value. To address this limitation, investigators employ stochastic microsimulation models, which simulate individual patient trajectories and enable more robust assessments of cost-effectiveness. As the field progresses from AI to extended intelligence and, ultimately, intelligent assistance, the role of humans becomes increasingly central. Rather than replacing clinical judgment, AI should augment human expertise, with healthcare professionals serving as the critical link between technological innovation and meaningful clinical benefit. This review examines whether AI applications in GI diseases can be implemented cost-effectively in routine clinical practice for diagnosis and treatment. It further explores the indispensable human dimension of AI adoption, emphasizing accountability, reliability, ethical oversight, and responsible implementation as essential safeguards for current and future clinical applications.

## 1 Introduction

Nearly two millennia ago, St. Paul the Apostle wrote in his First Epistle to the Corinthians: “*All things are lawful for me, but not all things are beneficial*.” Although rooted in the theological context of early Christianity, this statement conveys a timeless principle: the mere availability of an action does not necessarily justify its adoption. While St. Paul referred to the distinction between what was permissible and what was spiritually edifying, the same concept remains highly relevant in contemporary medicine, where technological innovation increasingly challenges the balance between capability and clinical value (The First Epistle to the Corinthians, n.d). Modern healthcare has become progressively influenced by consequentialist thinking, in which the value of an intervention is judged primarily by its outcomes.

The application of artificial intelligence (AI) in medicine has evolved remarkably over the past five decades. Early clinical decision-support systems—including Internist-I, MYCIN, and DXplain—represented pioneering attempts to assist physicians by integrating clinical information into computer-assisted diagnostic reasoning. Since the emergence of machine learning (ML), AI has expanded rapidly across virtually every domain of medicine, fundamentally transforming diagnostic and therapeutic approaches (Miller et al., 1982; Shortliffe, 1977; Barnett et al., 1987; Basics of Machine Learning, 2024).

Among medical specialties, gastroenterology has experienced one of the fastest rates of AI adoption. Advances in endoscopy and medical imaging have led to increasingly sophisticated computer-assisted systems capable of improving the detection of neoplastic and preneoplastic lesions while supporting diagnostic characterization and therapeutic decision-making. Recognizing this rapid evolution, international gastroenterology societies have published evidence-based position statements and clinical practice guidelines to facilitate the safe and effective integration of AI into routine clinical practice (Table 1) (Sultan et al., 2025; Messmann et al., 2022; Fujishiro et al., 2026; Mori et al., 2025; ASGE AI Task Force et al., 2025; Mori et al., 2023; Gross et al., 2026). The scientific literature reflects this extraordinary growth. Publications addressing AI in gastroenterology have increased almost exponentially over the past decade, accompanied by dedicated journal issues, specialized academic institutes, consensus statements, educational initiatives, and textbook chapters. Collectively, these developments illustrate the growing expectation that AI will become an integral component of future gastrointestinal (GI) practice (Shung and Iacucci, 2025; Chang et al., 2023; Artificial Intelligence Institute for Gastroenterology, n.d; Sood et al., 2024). The use of only “*Artificial Intelligen*ce” and “*Gastroenterolog*y” as Medical Subject Headings (MeSH) in the PubMed® authoritative search engine, clearly, reveals a nearly exponential increasing trend of corresponding bibliography over the last decades (Figure 1).

**Table 1:** Position statements/practice guidelines on AI on behalf of international gastroenterology governing bodies and endoscopic societies published over the last few years.

Governing bodies	Year of publication	Position statements/practice guidelines
ACG	2026	Artificial Intelligence for Gastroenterology Practice
WEO	2026	Position Statements for AI in Endoscopic Diagnosis of Gastric Epithelial Neoplasia
AGA	2025	Living Clinical Practice Guideline on Computer-Aided Detection-Assisted Colonoscopy
ASGE	2025	Consensus statements on the current landscape of AI applications in endoscopy, addressing roadblocks, and advancing artificial intelligence in gastroenterology
JGES	2025	AI in Gastrointestinal Endoscopy
WEO	2023	Benefits and challenges in implementation of AI in colonoscopy
ESGE	2022	Expected value of AI in gastrointestinal endoscopy

ACG: American College of Gastroenterology; WEO: World Endoscopy Organization; AGA: American Gastroenterological Association; ASGE: American Society for Gastrointestinal Endoscopy; JGES: Japan Gastroenterological Endoscopy Society; ESGE: European Society of Gastrointestinal Endoscopy; AI: Artificial Intelligence.

**Figure 1:**
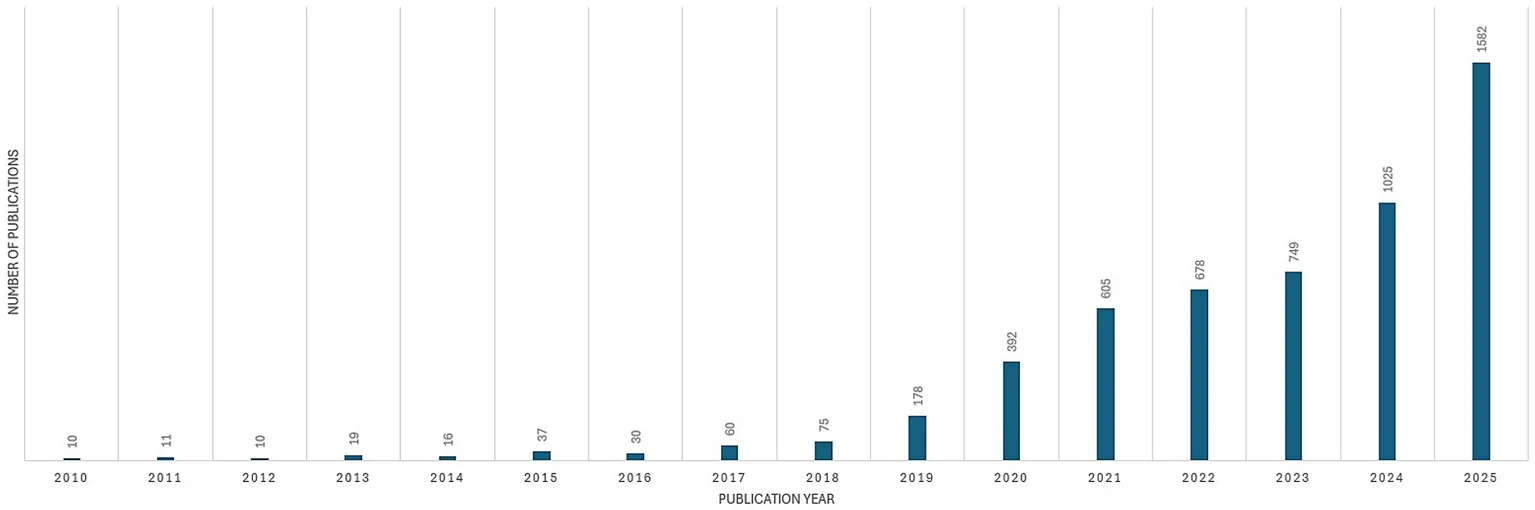
Number of manuscripts regarding AI in Gastroenterology published from 2010 in PubMed®. https://pubmed.ncbi.nlm.nih.gov/?term=artificial%20intelligence%2C%20gastroenterology&sort=date&timeline=expanded. Assessed August 31, 2026.

However, this unprecedented expansion has also created what may aptly be described as a *“data tsunami”* or *“datanami”*, an overwhelming and continuously expanding body of evidence that increasingly challenges clinicians’ and researchers’ ability to critically evaluate, synthesize, and translate new knowledge into everyday clinical practice (Faulx, 2025). On the other side, it marks the perplexity of publications, as the rapidly increasing volume of data generated in the modern digital age, often, exceeds the ability of researchers to manage, process, or analyse it effectively. This, eventually, reflects to an increased burden on the side of the endoscopist/treating physician when choosing the correct treatment plan for the patient implementing all this new information in everyday clinical practice.

This review examines critical aspects of economic AI evolution in GI healthcare and tries to report on frameworks through which it should be interpreted. By reporting on worldwide differences regarding reimbursement of AI application on everyday clinical practice, we aim to present data from microsimulation studies regarding the cost-effectiveness of AI-assisted technologies in contemporary gastroenterology research highlighting defaults that are, inevitably, part of an inherent methodologic bias. We, finally, explore the indispensable human dimension of AI adoption, emphasizing accountability, reliability, ethical oversight, and responsible implementation as essential safeguards for current and future clinical applications, so as to balance, for now, any discrepancies between research data and real-world efficacy reports.

## 2 The problem

Substantial investments have been directed toward the development and implementation of AI, driven by expectations of improved efficiency, enhanced diagnostic performance and reduced healthcare costs. Numerous systematic reviews and meta-analyses have consistently demonstrated that AI improves the detection of precancerous and malignant GI lesions and enhances diagnostic accuracy across a broad spectrum of GI disorders (Gangwani et al., 2026; Parkash et al., 2022; Barlow et al., 2026; Ahuja et al., 2026; Ebigbo et al., 2025; Spadaccini et al., 2024; Sedano et al., 2025; Kröner et al., 2021; Halvorsen and Mori, 2025).

Despite these encouraging findings, important limitations remain. Current AI systems continue to face challenges related to geographic bias in training datasets, regulatory approval, variability in real-world clinical performance, dependence on procedural quality, limited generalizability across healthcare settings, ethical concerns regarding patient privacy and algorithmic accountability, the concentration of AI development among a small number of technology companies and the integration of multimodal clinical data into sustainable implementation frameworks. Addressing these challenges is essential if AI is to achieve widespread clinical adoption while maintaining patient safety, transparency, and ethical integrity (Gong et al., 2025; Gadour et al., 2026).

Perhaps the most important unresolved issue, however, is economic rather than technological. AI systems require substantial investments in infrastructure, data acquisition, annotation, storage, maintenance, software updates, cybersecurity, regulatory compliance, and personnel training. Consequently, AI may not reduce healthcare expenditure but, instead, redistribute costs from traditional clinical resources to digital infrastructure. Whether this transition ultimately represents good value for healthcare systems remains uncertain. In such a complex AI environment, where human-computer interaction is governed by variables that can distort research data, barriers of AI implementation in clinical practice still need to be overcome, and, more importantly, the human factor (either the patient or the treating physician/endoscopist) needs to be set as the highest priority (Ye et al., 2025).

Accordingly, the central question is no longer whether AI improves diagnostic accuracy—it, clearly, does in many applications—but whether these improvements translate into clinically meaningful benefits that justify the associated costs. In other words, can improved lesion detection ultimately reduce morbidity, mortality, and healthcare expenditure sufficiently to warrant routine implementation? Answering this question requires robust methods for measuring value. The endorsement of any diagnostic or therapeutic innovation should depend not only on technical performance but also on objective measures of clinical effectiveness, patient outcomes, and economic sustainability. Therefore, determining the cost-effectiveness of AI-assisted technologies has become one of the major challenges facing contemporary gastroenterology research.

## 3 Economic evaluation in health care

Health systems operate within finite financial resources, making economic evaluation an essential component of healthcare decision-making. Before a new diagnostic technology or therapeutic intervention is incorporated into routine clinical practice, policymakers must determine whether its additional clinical benefits justify its additional costs. Cost-effectiveness analysis (CEA) provides the principal framework for addressing this question by comparing the incremental costs and health outcomes of competing healthcare strategies (Mitchell, 2021). The National Institute for Health and Care Excellence (NICE) has adopted a guidance under which researchers can measure and value health effects in cost-utility analyses (Measuring and Valuing Health Effects in Cost-Utility Analyses, n.d).

The most widely accepted measure of cost-effectiveness is the Incremental Cost-Effectiveness Ratio (ICER), which represents the additional cost required to achieve one additional unit of health benefit compared with an alternative strategy. Costs typically include expenditures related to diagnostic procedures, medications, hospitalizations, follow-up care and management of complications, whereas health outcomes are most commonly expressed as Quality-Adjusted Life Years (QALYs), a metric that combines both survival and health-related quality of life into a single measure (Sultan et al., 2025; Messmann et al., 2022; Fujishiro et al., 2026). Another widely used population-level measure is the Disability-Adjusted Life Year (DALY), which quantifies the overall burden of disease by combining years of life lost because of premature mortality with years lived with disability. DALYs are particularly useful for evaluating the societal value of healthcare interventions and informing research prioritization (Drummond, 2005; Thomas and Chalkidou, 2016; Woods et al., 2020).

In everyday clinical practice, physicians are generally more familiar with measures of treatment efficacy such as Relative Risk (RR) and the Number Needed to Treat (NNT). RR estimates the relative probability of an outcome occurring in the intervention group compared with the control group, whereas NNT represents the number of patients who must receive an intervention for one additional patient to benefit. Although these measures provide valuable information regarding clinical effectiveness, they do not incorporate economic considerations and, therefore, cannot determine whether an intervention represents good value for healthcare systems (Tripepi et al., 2007; Irwig et al., 2008).

Consequently, while RR and NNT help clinicians evaluate efficacy, healthcare policymakers increasingly rely on ICERs and QALYs when making reimbursement and implementation decisions. This distinction is particularly relevant in AI research, where improvements in diagnostic performance do not, necessarily, translate into cost-effective healthcare delivery. Although AI has consistently demonstrated improvements in diagnostic performance, the evidence supporting its economic value remains comparatively limited. Understanding this discrepancy requires careful examination of how efficacy has been measured in published studies.

### 3.1 Efficacy of AI in gastroenterology studies

Most studies report measures of diagnostic accuracy, adenoma detection rate (ADR) in lower GI endoscopies, sensitivity, specificity or RR rather than formal health-economic outcomes, such as ICERs or QALYs. This emphasis reflects the current role of AI as a clinical decision-support tool rather than an independent therapeutic intervention. In endoscopy, AI functions as a “second observer,” assisting endoscopists by highlighting suspicious lesions and improving lesion characterization instead of replacing physician judgment. Consequently, investigators generally evaluate AI by measuring improvements in lesion detection or reductions in missed lesions rather than by calculating traditional therapeutic metrics such as the NNT (El-Sayed et al., 2025).

Although improvements in ADR have been consistently demonstrated, the fundamental clinical question remains unresolved: do these diagnostic gains ultimately translate into fewer colorectal cancers (CRCs), lower mortality, improved quality of life (QoL), and sustainable healthcare savings? This distinction is crucial. A statistically significant increase in lesion detection does not, necessarily, imply meaningful improvements in patient-centred outcomes. This holds especially true as extra lesions found with computer-aided detection (CADe) are mostly diminutive, 5 mm or less. Meta-analyses have not established a clear benefit for advanced adenoma detection, and the effect on sessile serrated lesions is inconsistent (Soleymanjahi et al., 2024). Increased detection may also result in higher numbers of surveillance procedures, additional polypectomies, greater pathology workload, prolonged procedure times, and increased healthcare expenditure. Makar et al., reported a 39% increase in the rate of non-neoplastic resection (RR, 1.39; 95% CI, 1.23–1.57; *p* < 0.001; Makar et al., 2025). Consequently, improved diagnostic performance should not automatically be interpreted as evidence of clinical value.

Recognizing this limitation, recent methodological initiatives have sought to improve the quality of economic reporting in AI research. The Consolidated Health Economic Evaluation Reporting Standards for AI (CHEERS-AI) provide a structured framework for conducting and reporting economic evaluations of AI-based healthcare interventions. By promoting transparent reporting of costs, modelling assumptions, uncertainty analyses, and AI-specific methodological considerations, CHEERS-AI aims to facilitate evidence-based reimbursement decisions and improve comparability among studies (Elvidge et al., 2024). This is of paramount importance as healthcare interventions using AI have serious methodological flaws without reporting on proper economic evaluations (EEs), rendering reimbursement decisions even more ambiguous (Vithlani et al., 2023).

Similarly, the QUAIDE framework has been developed to improve the design, conduct, and reporting of preclinical AI studies in diagnostic endoscopy. Standardized methodological frameworks such as CHEERS-AI and QUAIDE are likely to play an increasingly important role as AI technologies progress from proof-of-concept studies toward routine clinical implementation (Antonelli et al., 2024). Ultimately, the future success of AI in gastroenterology will depend not only on achieving superior diagnostic accuracy but, also, on demonstrating measurable improvements in patient outcomes, healthcare efficiency, and cost-effectiveness.

### 3.2 Reimbursement

The clinical adoption of AI is closely linked to reimbursement policy. Regardless of technological performance, widespread implementation is unlikely unless healthcare systems establish sustainable reimbursement mechanisms that adequately compensate providers for the use of AI-assisted technologies (Wu et al., 2023; Abràmoff et al., 2022). Reimbursement policies vary substantially across healthcare systems, reflecting differences in economic resources, healthcare priorities, reimbursement structures and national health technology assessment (HTA) processes. Consequently, the cost-effectiveness of AI cannot be interpreted independently of the healthcare environment in which it is implemented (O'Rourke et al., 2020; Turner et al., 2021; Gu et al., 2022; Alshaikh et al., 2025).

The current international landscape illustrates this heterogeneity. In the United States, although several AI-assisted endoscopic systems have received regulatory approval, no universal Medicare reimbursement exists specifically for AI-assisted colonoscopy. Hospitals generally absorb AI-related costs within existing reimbursement structures, while private insurers negotiate reimbursement individually (ASGE AI Task Force et al., 2025; Sun and Littenberg, 2025). Across Europe, reimbursement remains heterogeneous. Germany has established advanced pathways for digital health technologies, although these rarely include AI-assisted endoscopy. France and the United Kingdom (UK) continue to evaluate reimbursement models, with purchasing decisions frequently delegated to individual hospitals or regional healthcare authorities (Farah et al., 2023). Japan currently represents the most advanced example, having introduced national reimbursement for AI-assisted CADe, whereas China and Australia have yet to establish dedicated reimbursement mechanisms (Mori et al., 2025; Yasuharu et al., 2025).

Sun and Littenberg reported on the utilization of Centers for Medicare and Medicaid Services (CMS) in determining the reimbursement landscape for AI-driven medical innovations (Sun and Littenberg, 2025). However, only a small number of AI devices have achieved reimbursement via CMS via AI-specific common procedural terminology codes and new technology add-on payments (El-Sayed et al., 2025).

### 3.3 Microsimulation models: the bridge between diagnostic performance and clinical value

Traditional clinical trials are well suited to evaluating short-term diagnostic performance but are considerably less effective in assessing the long-term clinical and economic consequences of AI. Outcomes such as CRC incidence, cancer-related mortality, healthcare costs, and QALYs may not become apparent for many years after implementation, making prospective randomized trials (RCTs) impractical, time-consuming, and prohibitively expensive.

For this reason, stochastic microsimulation models have emerged as the preferred methodological approach for evaluating the long-term value of AI-assisted healthcare interventions. Rather than analysing populations as homogeneous groups, microsimulation models simulate the clinical trajectory of individual patients, incorporating demographic characteristics, disease progression, screening participation, diagnostic accuracy, treatment pathways, surveillance intervals, competing mortality, healthcare costs, and QoL outcomes. Repeating these simulations across hundreds of thousands of virtual patients allows investigators to estimate the long-term consequences of alternative screening and management strategies with a level of detail that cannot be achieved in conventional clinical trials. Although characterized by inherent limitations (i.e., assumptions on the patient behaviour, acceptance, and implementation of screening programs), their endpoint is to calculate ICERs so as to guide healthcare systems into whether a real-time AI application should be applied in endoscopic procedures (Buskermolen et al., 2018; Microsimulation Models to Inform Colorectal Cancer Screening Decisions, n.d; Gini et al., 2021; Rao et al., 2022; El Arab and Al Moosa, 2025).

Unlike conventional decision-tree analyses, microsimulation models account for uncertainty, competing risks, recurrent events, and patient heterogeneity, thereby providing a more realistic representation of clinical practice. Consequently, they have become an indispensable component of contemporary health technology assessment and healthcare policy development. Microsimulation models for AI are heavily focused on three primary areas: (i) CRC Screening, (ii) Barrett’s Esophagus (BE) and Esophageal Adenocarcinoma (EAC) and (iii) Gastric Cancer (GC). Parameters used in a microsimulation model to inform CRC screening decisions involve demographics, natural history and screening (Buskermolen et al., 2018; Microsimulation Models to Inform Colorectal Cancer Screening Decisions, n.d; Gini et al., 2021; Rao et al., 2022; El Arab and Al Moosa, 2025).

#### 3.3.1 Literature identification and approach

This manuscript was designed as a structured narrative review aimed at synthesizing contemporary evidence on the efficacy of AI in GI endoscopy. Given the broad scope of the topic and the reported heterogeneity of the studies, a narrative synthesis approach was selected rather than a formal systematic review to integrate clinical data across heterogenous study designs. A comprehensive literature search was conducted using major biomedical databases, including Pubmed/MEDLINE, Embase and Scopus, to identify relevant studies published in English. Eligible studies included those evaluating microsimulation models reporting on the cost effectiveness of AI in gastrointestinal endoscopy. Search terms included combinations of controlled vocabulary and free-text keywords such as “artificial intelligence,” “microsimulation,” “gastroenterology,” “endoscopy,” “efficacy,” “cost effectiveness,” “colonoscopy,” and “esophagogastroduodenoscopy.” Reference lists of relevant reviews, meta-analyses, and guideline documents were manually screened to identify additional pertinent studies not retrieved in the primary database search. Studies were selected based on their relevance to the clinical aspects of AI efficacy in GI endoscopy. Data extraction was performed through iterative review of the selected literature, focusing on key domains predefined according to the conceptual framework of the manuscript. Case reports, small uncontrolled studies, and studies with limited methodological rigor as well as those not focused on microsimulation models were excluded. As a narrative review, this study did not involve primary data generation, patient recruitment or statistical pooling results.

Table 2 summarizes studies using microsimulation methodology for measuring cost effectiveness of AI in GI endoscopy (7 for AI in colonoscopies, 1 for AI in BE and 2 for AI in esophagogastroduodenoscopies). The available evidence derived from microsimulation studies is generally encouraging. All but one reported on dominant (cost-saving) or cost-effective strategies when AI is incorporated in clinical practice compared with conventional endoscopy (standardized economic conclusion considering a 2026 USD Baseline), although the magnitude of benefit varies according to the assumptions incorporated into each model (Barkun et al., 2023; Areia et al., 2022; Tan et al., 2026; Sekiguchi et al., 2023; Halvorsen et al., 2025; Bustamante-Balén et al., 2025; Libanio et al., 2024; Thiruvengadam et al., 2023; Yonazu et al., 2023; Wong et al., 2025).

**Table 2:** Studies using microsimulation methodology to measure cost effectiveness of AI in gastrointestinal endoscopy.

Study	Publication date	Methodology used	Population	Comparators	Outcome	Standardized WTP (2026 USD Baseline)	Standardized Economic Conclusion (2026 USD Baseline)	Conclusion
Barkun et al.	2023	Markov microsimulation model with 1-year cycles and a lifetime horizon	Canadian health care perspective	ICER comparing CADe to conventional colonoscopy polyp detection amongst patients with a positive FIT	Life years and QALY gains for the CADe and conventional colonoscopy groups were $19.144 vs. $19.125 and $17.137 vs. $17.113, respectively. CADe and conventional colonoscopies’ overall per-case costs were $2990.74 and $3004.59, respectively. ICER dominant for both outcomes, showing that CADe colonoscopy is cost-effective. CADe strategy was cost-effective in up to 73.4% of scenarios	$54,500	Dominant (Cost-Saving). Higher QALYs with lower total per-case costs	The addition of CADe solution to colonoscopy is a dominant, cost-effective strategy when used in FIT-positive patients
Areia et al.	2022	Markov model microsimulation every 10 years starting at age 50 years and finishing at age 80 years, with follow-up until age 100 years (primary analysis) and once-in-life screening colonoscopy at age 65 years (secondary analysis)	Hypothetical cohort of 100.000 individuals in the USA aged 50–100 years.	Colonoscopy with and without AI for CRC screening for individuals at average risk (no personal or family history of CRC, adenomas, IBD, or hereditary CRC).	In the primary analyses, compared with no screening, the relative reduction of CRC incidence with screening colonoscopy without AI tools was 44.2% and with screening colonoscopy with AI tools was 48.9% (4.8% incremental gain). Compared with no screening, the relative reduction in CRC mortality with screening colonoscopy with no AI was 48.7% and with screening colonoscopy with AI was 52.3% (3.6% incremental gain). AI detection tools decreased the discounted costs per screened individual from $3.400 to $3.343 (a saving of $57 per individual). Results were similar in the secondary analyses modelling once-in-life colonoscopy. AI detection during screening colonoscopy resulted in yearly additional prevention of 7.194 CRC cases and 2.089 related deaths, and a yearly saving of $290 million.	$114,800	Dominant (Cost-Saving). Saves approx. $65 per screened person.	Implementation of AI detection tools in screening colonoscopy is a cost-saving strategy to further prevent CRC incidence and mortality
Tan et al.	2026	Markov microsimulation model in patients with NDBE undergoing surveillance from age 50 to 80 years, with follow-up until age 100	1.000 Australian individuals	AI-assisted surveillance with targeted biopsies against standard endoscopy with four-quadrant biopsies under 3-yearly and 5-yearly surveillance	AI-assisted surveillance was cost effective across both intervals, with ICERs of $14. 039/QALY (3-yearly) and $3.609/QALY (5-yearly). Compared with standard surveillance, AI reduced the cumulative incidence of advanced EAC by 5 and 3 cases per 1,000 people (relative reductions of 3.5 and 1.6%) for 3- and 5-yearly surveillance, respectively. Conversely, AI increased HGD/T1 detection by 27 and 38 cases per 1,000 people (relative increases of 21.8 and 27.7%) for 3- and 5-yearly surveillance, respectively. AI reduced missed HGD/T1 incidence by 37 and 45 cases per 1,000 people (relative reductions of 72.9 and 72.2%) for 3- and 5-yearly surveillance, respectively.	$36,500	Borderline Cost-Effective (~$27,800/QALY).	Highly sensitive to the risk of progression. AI-assisted endoscopic surveillance in BE was a cost-effective strategy in the Australian health care setting, reducing the cumulative incidence of advanced EAC and missed HGD/T1 lesions.
Sekiguchi et al.	2023	Monte Carlo simulation	Japanese individuals aged 40–74 years	Cost-effectiveness of colonoscopy with and without CADe for a population aged 40–74 years under CRC screening with a FIT	Compared to conventional colonoscopy, CADe colonoscopy showed a higher QALY (20.4098 vs. 20.4088) and lower CRC incidence (2,373 vs. 2,415 per 100,000) and mortality (561 vs. 569 per 100.000). When the CADe cost was set at JPY1.000–6.000, the ICER per QALY gained for CADe colonoscopy was lower than JPY 5.000.000 (JPY 796.328–4.971.274). The CADe cost threshold at which the ICER for CADe colonoscopy exceeded JPY 5.000.000 was JPY 6.040.	$42,500	Cost-Effective (~$6,200 to $38,500/QALY) if the AI per-use fee is kept below $47.	Computer-aided detection systems for colonoscopy has the potential to be cost-effective when the CADe cost is up to JPY 6.000.
Halvorsen et al.	2025	Markov microsimulation model of 10-year life histories for individuals who were screened with one colonoscopy or by colonoscopy after FIT screening every other year with a threshold for colonoscopy of 20 μg Hb/g faeces	Four cohorts of 100.000 European individuals aged 60–69 years	The intervention was one screening of colonoscopy and a screening of colonoscopy after FIT every other year with CADe. The comparison group had the same screening every other year without CADe	824 (0.82%) diagnosed with CRC within 10 years without CADe versus 713 (0.71%) with CADe [risk difference −0.11% (95% CI-0.43 to 0.21%)]. For FIT screening colonoscopy, the risk was 5.82% (*n* = 5,820) without CADe versus 5.77% (*n* = 5,770) with CADe [difference −0.05% (−0.33 to 0.15%)]. The risk of surveillance colonoscopy increased from 26.45% (*n* = 26.453) to 32.82% (*n* = 32.819) [difference 6.37% (5.8 to 6.9%)] for colonoscopy screening and from 52.26% (*n* = 52.263) to 53.08% (*n* = 53.082) [difference 0.82% (0.38 to 1.26%)) for FIT screening colonoscopy.	$55,000	Borderline/Non-Dominant. Due to high surveillance costs from small polyps, it is likely close to the WTP line.	With low certainty of evidence, adoption of CADe in population-based screening provides small and uncertain clinical meaningful benefit, no incremental harms, and increased surveillance burden after screening
Bustamante-Balén et al.	2025	Markov microsimulation model designed to estimate total costs, life years gained (LYG), and QALYs over a lifetime horizon with annual cycles	A hypothetical cohort of 1.000 patients eligible for colonoscopy (mean age 61.32 years) from a Spanish National Health System perspective	Detected polyp management involved polypectomy and histopathology in standard practice, whereas with CADe/CADx leave-in-situ strategy was applied for ≤ 5 mm rectosigmoid non-adenomas and resect-and-discard strategy for the rest of ≤ 5 mm polyps	CADe/CADx was more effective (16.37 LYG and 14.32 QALYs) than standard practice (16.33 LYG and 14.27 QALYs) over a lifetime horizon. Total cost per patient was €2,300.76 with CADe/CADx and €2,508.75 with colonoscopy alone. In a hypothetical cohort of 1,000 patients, CADe/CADx avoided 173 polypectomies, 370 histopathologies, and 7 CRC cases	$34,200	Dominant (Cost-Saving). Offsets biopsy costs via long-term cancer prevention.	CADe/CADx would result in a dominant strategy versus standard practice in patients undergoing colonoscopy in Spain
Wong et al.	2025	Markov microsimulation study of a hypothetical population consistent with current clinical practice in Asia, where screening typically begins at the age of 50 years	A hypothetical population of 100,000 average-risk individuals aged 50 years with no history or symptoms of CRC	Hypothesized that the entire population received four distinct screening strategies, including (1) FIT followed by conventional colonoscopy if the FIT result is positive (FIT+colonoscopy), (2) FIT followed by AI-assisted colonoscopy if the FIT result is positive (FIT+AI-assisted colonoscopy), (3) colonoscopy as the primary screening test, and (4) AI-assisted colonoscopy as the primary screening test. These screening participants were followed up until the age of 75 years. These strategies were compared to a no screening scenario	Compared to no screening strategy, the ICERs of FIT+colonoscopy (FIT followed by conventional colonoscopy if the FIT result is positive), FIT+AI-assisted colonoscopy (FIT followed by AI-assisted colonoscopy if the FIT result is positive), colonoscopy alone, and AI-assisted colonoscopy were US $138,539, US $122,539, US $203,929, and US $180,444, respectively. When compared with FIT+colonoscopy, the FIT+AI-assisted colonoscopy strategy resulted in fewer cancer-related life years lost (5,355 y vs. 5,327 y), a higher number and proportion of CRC cases prevented (120 vs. 132 and 3.7% vs. 4.1%), more life years saved (280 y vs. 308 y), and lower total cost per life year saved (US $944,008 vs. US $854,367). FIT+AI-assisted colonoscopy, which had the lowest ICER (US $122,539) dominated all other strategies, particularly compared to FIT+colonoscopy, with an ICER of –US $36,462	$51,300	Dominant (Cost-Saving) when added to FIT-positive tracks (saves ~$38,400 over routine)	For an Asian population, FIT followed by AI-assisted colonoscopy represented the most cost-effective CRC screening strategy. It had the lowest ICER and the lowest additional cost among all 4 evaluated strategies
Libanio et al.	2024	Markov microsimulation model for GC detection in settings with different GC incidence and different accuracies of AI systems	Netherlands, Italy and Portugal	Comparing different screening strategies (no screening versus single EGD) at 50 years versus stand-alone EGD every 5/10 years versus combined EGD and screening colonoscopy once or twice per decade	Without AI, one single EGD at 50 years (Netherlands, Italy, Portugal), EGD combined with screening colonoscopy once per decade (Italy and Portugal) and EGD combined with screening colonoscopy twice per decade (Portugal) are cost-effective when compared with no screening. If AI increases the accuracy of EGD by at least 1% in comparison to the accuracy WLE (89%), combined screening twice per decade also becomes cost-effective in Italy. If AI accuracy reaches at least 96%, combined screening once per decade is also cost-effective in the Netherlands	$56,400	Cost-Effective (~$20,800/QALY) for combined multi-organ gastric/colorectal screening track.	In European countries, AI-assisted EGD may improve the cost-effectiveness of GC screening with combined EGD and screening colonoscopy
Thiruvengadam et al.	2023	Semi-Markov microsimulation model simulating a base case scenario of no CRC screening; patients were followed up until age 100 or death	Across USA states	Modelled screening colonoscopy beginning at age 45. If no polyps were detected, a 10-year interval was used for screening colonoscopy. Patients who had a polyp detected and removed were transitioned to surveillance colonoscopy. A 7-year post-polypectomy surveillance interval was used after removing <5-mm tubular adenomas and 5- to 10-mm tubular adenomas and a 3-year interval for advanced adenomas. Patients underwent CRC screening until age 75 and CRC surveillance until age 80. In patients in both the natural history module and the colonoscopy arms, postoperative colonoscopy surveillance colonoscopy was performed at years 1, 3, and 5 in patients with localized and regional CRC that was resected	When modelling CAD improved ADR for all endoscopists, the CAD cohort had 79 and 34 fewer lifetime CRC cases and deaths, respectively, per 10,000 persons. This scenario was dominant with a cost savings of $143 and incremental effectiveness of 0.01 QALYs. Threshold analysis demonstrated that CAD would be cost-effective up to an additional cost of $579 per colonoscopy, or if it increases ADR from 26% to at least 30%. CAD reduced CRC incidence and mortality when limited to improving ADRs for low-ADR endoscopists (ADR < 25%), with 67 fewer CRC cases and 28 CRC deaths per 10,000 persons compared with traditional colonoscopy	$111,200	Highly Cost-Effective (~$6,950/QALY). Remains stable across sensitivity loops.	CAD needs to improve mean ADR from 26% to at least 30% or cost less than $579 per colonoscopy to be cost-effective when compared with traditional colonoscopy
Yonazu et al.	2023	Decision trees with Markov models were built	Japanese cohort	Analyse the ICER of using CADx relative to the pre-AI status quo in patients with moderate to severe gastritis caused by HP infection	The ICER of CADx was forecasted to be $11,093/QALY. The sensitivity analyses demonstrated that the expected ICERs were within the willingness-to-pay threshold of $50,000/QALY when the cost of the CAD was less than $104	$52,100	Cost-Effective (~$11,850/QALY) only if the per-case system marginal cost stays under $104.	CADx for EGCs may decrease their misdiagnosis, contributing to improved cost-effectiveness

ICER: Incremental Cost-Effectiveness Ratio; CADe: computer-aided detection; QALYs: Quality-Adjusted Life Years; AI: Artificial Intelligence; CRC: colorectal cancer; IBD: inflammatory bowel disease; NDBE: nondysplastic Barrett’s esophagus; HGD: high grade dysplasia; FIT: faecal immunochemical test; JPY: Japanese Yen: BE: Barrett’s esophagus; EAC: esophageal adenocarcinoma; LYG: life years gained; CADx: computer-assisted diagnosis; EGD: esophagogastroduodenoscopy; GC: gastric cancer; WLE: white-light endoscopy: USA: United States of America; CAD: computer-aided detection; ADR: adenoma detection rate; EGC: early gastric cancer; HP: Helicobacter pylori; WTP: Willingness-to-Pay.

Methodological data of the microsimulation studies reporting cost effectiveness of AI in gastrointestinal endoscopy taken into consideration when accounting for their quality are depicted in Table 3. The quality of microsimulation studies of AI in GI endoscopy using the CHEERS-AI checklist framework is depicted in Figure 2.

**Table 3:** Methodological data of the microsimulation studies reporting cost effectiveness of AI in gastrointestinal endoscopy taken into consideration when accounting for their quality.

Study	Time horizon	Discount rate	Probabilistic sensitivity analysis	Funding source and COI profile
Barkun et al.	Lifetime	1.5–3.0%	Yes	Academic institutional funding. COI: Authors report consultant/advisory roles for medical device companies
Areia et al.	50–100 years	3.0%	Yes	Supported in part by European research funds. COI: Some co-authors received honoraria/lecture fees from Fujifilm and Medtronic
Tan et al.	Up to 100 years	5.0%	Yes	Hospital & University Departmental funding (Adelaide). COI: Declared free of any commercial conflicts of interest.
Sekiguchi et al.	40–74 years	2.0%	Yes	National Cancer Center Research Fund (Japan). COI: Authors declare no commercial conflicts of interest.
Halvorsen et al.	10 years	3.0%	Yes	European Union Horizon Europe Research Grant. COI: Purely academic investigators, independent of industry; zero COI.
Bustamante-Balén et al.	Lifetime	3.0%	Yes	Spanish Gastroenterological Society Grant. COI: Some authors reported lecture fees or consulting work with Olympus.
Wong et al.	Lifetime	3.0%	Yes	Government Health and Medical Research Fund. COI: Academic researchers with no industry-specific commercial conflicts.
Libanio et al.	Lifetime	3.0%	Yes	ESGE. COI: Purely academic investigators with no commercial ties.
Thiruvengadam et al.	Lifetime	3.0%	Yes	NIH Grants. COI: Certain co-authors serve as scientific advisors to medical AI startups.
Yonazu et al.	3 years	2.0%	Yes	Private Corporate Funding (AI Medical Service Inc.). COI: High Risk. Key authors (CEO/CTO) are executives of the developer company.

COI: Conflicts of Interest; ESGE: European Society of Gastrointestinal Endoscopy; NIH: National Institutes of Health; AI: artificial intelligence; CEO/CTO: Chief Executive Officer/Chief Technology Officer.

**Figure 2:**
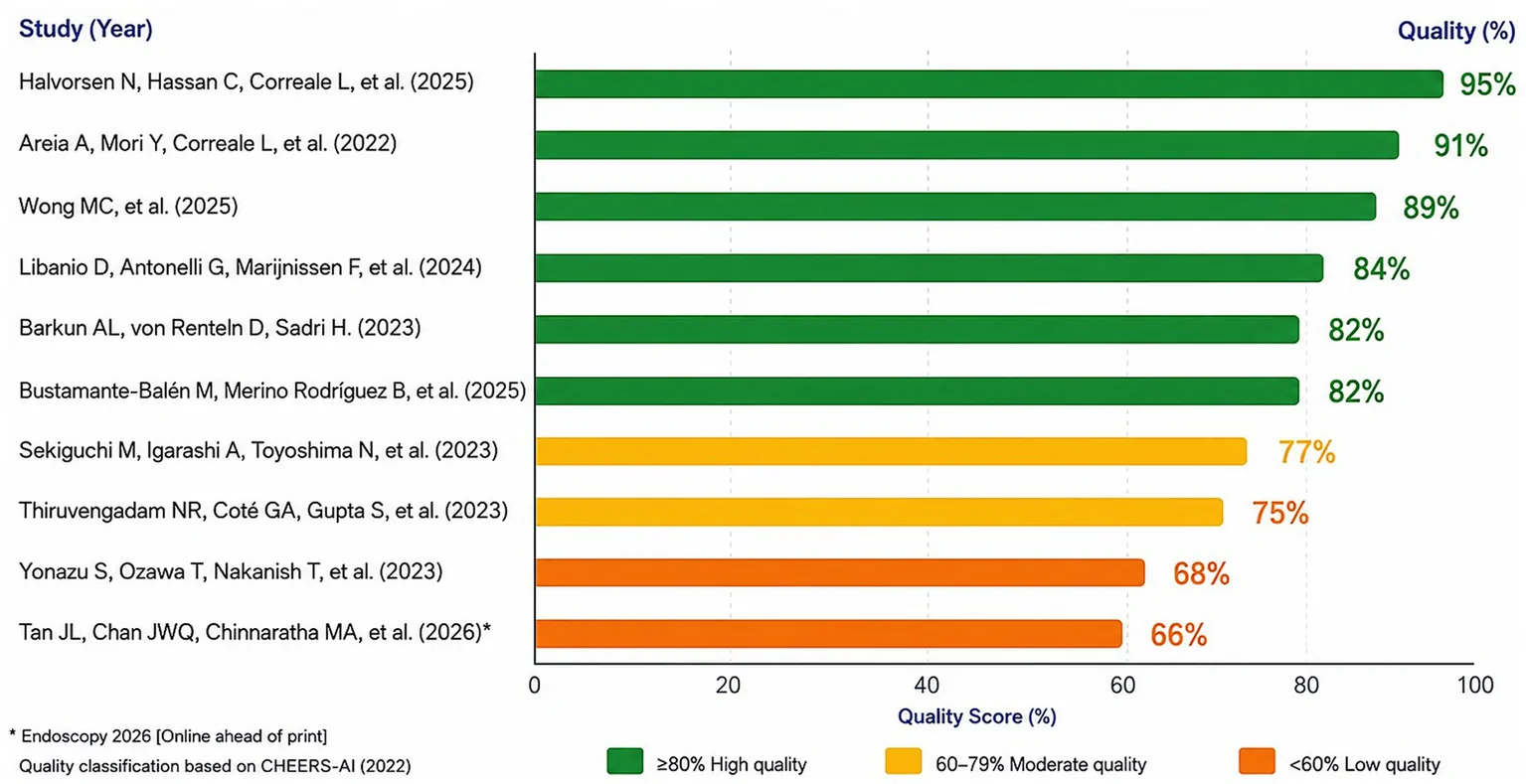
The quality of microsimulation studies of AI in GI endoscopy using the CHEERS-AI checklist framework.

### 3.4 Inherent methodologic bias

Although advantageous, except from being mostly concentrated in lower endoscopy data as noted before, these microsimulation studies have methodological bias. Existing models frequently assume that AI performance remains constant over time. In reality, AI algorithms evolve continuously through software updates, retraining, and adaptation to new datasets, making traditional economic evaluations rapidly outdated. Moreover, relatively few studies incorporate indirect costs, including workforce training, software maintenance, cybersecurity, cloud computing infrastructure, medicolegal liability, and periodic regulatory compliance. These expenditures may substantially influence the true cost of AI implementation but are often omitted from current analyses. Finally, many models implicitly assume perfect integration of AI into clinical workflows. However, successful implementation depends on human acceptance, organisational culture, clinician training, interoperability with existing electronic health record systems, and patient trust—factors that remain difficult to quantify but are likely to influence real-world effectiveness (Buskermolen et al., 2018; Microsimulation Models to Inform Colorectal Cancer Screening Decisions, n.d; Gini et al., 2021; Rao et al., 2022; El Arab and Al Moosa, 2025).

Microsimulation models, inevitably, depend on assumptions regarding adenoma prevalence, lesion progression, diagnostic accuracy, patient adherence to screening and surveillance, procedure costs, healthcare utilization and long-term treatment outcomes. Variations in these assumptions may substantially influence model outputs and explain the differences observed among published economic evaluations. Besides that, most current models assume relatively stable improvements in adenoma detection rates following AI implementation. Whether these assumptions remain valid as AI systems evolve, endoscopists gain experience, and healthcare systems adapt to new technologies remains uncertain. Consequently, the conclusions generated by today’s models should be regarded as dynamic estimates rather than definitive predictions (Buskermolen et al., 2018; Microsimulation Models to Inform Colorectal Cancer Screening Decisions, n.d; Gini et al., 2021; Rao et al., 2022; El Arab and Al Moosa, 2025).

Furthermore, many published studies have evaluated AI under idealized conditions within organised screening programs (i.e., for CRC). Real-world implementation may produce different outcomes because of variations in operator expertise, patient populations, healthcare infrastructure, reimbursement systems, regulatory environments, and resource availability (Buskermolen et al., 2018; Microsimulation Models to Inform Colorectal Cancer Screening Decisions, n.d; Gini et al., 2021; Rao et al., 2022; El Arab and Al Moosa, 2025). This is exactly the rationale behind the fact that conclusions based on microsimulation and economic models should be interpreted cautiously, as their estimates depend on assumptions regarding AI performance, costs, adherence, and healthcare settings.

## 4 Why human expertise remains indispensable

Extended Intelligence (EI) is a human-centred framework that views technology as a partner designed to augment human cognition, rather than replace it. It shifts focus from AI competing with humans to a collaborative system where AI handles data processing and humans manage context, creativity, and ethics. Emerging from the interaction between users and AI systems, data-driven AI systems constitute a distinct psychological system (Abbosh et al., 2025; Chiriatti et al., 2024; Chacón et al., 2021; Nenna et al., 2023). AI should not be regarded as the ultimate objective of technological innovation in medicine. Rather, it represents one stage in the evolution of clinical decision support, progressing from automated data processing to systems capable of augmenting human reasoning. The future of AI in healthcare is therefore likely to be defined less by autonomous decision-making than by increasingly sophisticated forms of intelligent assistance (IA) that complement, rather than replace, clinical expertise (Abbosh et al., 2025; Chiriatti et al., 2024; Chacón et al., 2021; Nenna et al., 2023).

As these capabilities continue to evolve, the interaction between clinician and AI is becoming progressively collaborative rather than hierarchical. Accordingly, the future should not be framed as a competition between humans and machines but as the development of complementary partnerships in which computational intelligence and clinical judgment contribute distinct but equally essential strengths (Angulo et al., 2023; Grinin et al., 2024). A human-centred IA system in other aspects of social life and productivity has already been proposed; it is a matter of time before IA makes its mark in every clinical medical practice (Bechinie et al., 2024; Azvine et al., 2000).

Recent discussions surrounding the so-called 30% rule provide a useful framework for understanding the appropriate role of AI in clinical medicine. Although the precise numerical threshold should not be interpreted literally, the underlying principle is highly relevant: AI may perform a substantial proportion of repetitive analytical tasks, but responsibility for interpretation, contextual understanding, ethical judgment, communication, and final decision-making remains fundamentally human. Although not an established theory in the academic literature, the 30/70 split is a heuristic practical rule of thumb that advocates AI for repetitive work and measuring the results, while humans remain responsible for important judgment calls (What is the 30% rule in AI?, 2026a, b).

### 4.1 Moral responsibility in the era of AI

Perhaps the most profound challenge associated with AI implementation is not technological but ethical. Traditional medical ethics assumes that clinical decisions originate from identifiable healthcare professionals who can explain, justify, and accept responsibility for their actions. AI introduces additional layers of complexity because clinical recommendations increasingly emerge from interactions between algorithms, healthcare professionals, institutions, software developers, and regulatory frameworks. Responsibility, therefore, becomes distributed rather than individual.

Moral responsibility encompasses several complementary dimensions, including causal responsibility, role responsibility, capacity responsibility, virtue responsibility, and accountability. These dimensions should not be viewed as competing concepts but as interconnected components of responsible AI governance (Noy and Zhang, 2023).

Importantly, assigning responsibility to an AI system is philosophically problematic because AI possesses neither moral agency nor intentionality. Algorithms can generate recommendations, identify patterns, and estimate probabilities, but they cannot assume ethical responsibility for clinical decisions or their consequences. Accordingly, ultimate responsibility for patient care continues to reside with healthcare professionals and the institutions within which AI is deployed. This principle should remain unchanged regardless of future technological developments.

With all that said and done, it makes even more sense that humans [health professionals (physician and nurses), patients and informal caregivers] role] should be the X-factor, i.e., the link responsible for connecting AI and clinical benefit. This is of the outmost importance as the “responsibility gap” where no-one is fully accountable for AI-generated outcomes is, clearly, emerging as the next big issue that need to be resolved. Researchers have already identified two aspects of future concern: i) digital health technologies (amongst them AI) can expand and shift existing ‘role responsibilities’ among caregivers, patients and technology and ii) moral responsibility is often equated with liability or accountability, without clear justification (Meier et al., 2025).

Kwok *et al*., very recently reported on the Supportive Accountability Model (SAM), a theoretical framework designed to enhance adherence to Digital Health Interventions (DHIs), as seen in the case of AI, by incorporating structured human support. Their findings revealed that implementation of human support by peers and peer specialists, health experts and practitioners, trained coaches and members of the research study team varied in terms of delivery method, frequency and duration. As far as accountability was concerned, there was a lack of explicit focus on bond and legitimacy (Kwok et al., 2025). A recent survey on the perceptions of UK gastroenterologists and endoscopists to AI revealed exactly that; the vast majority of participants (85%) reported that one of the most significant challenge was accountability for incorrect diagnoses (Kader et al., 2022).

AI ethics addresses concerns arising from the development and implementation of new digital technologies. In an era of constant developments regarding AI that has yet to be regulated, data privacy, accountability, and bias are a matter of serious concern to academia, regulatory authorities, stakeholders and policy implementers. The immediate future suggests the incorporation of responsible AI principles as a vital safeguard, not only in curbing the frequency of ethical issues but also in minimizing their impact on patients’ wellbeing, especially as questions about its impact on cancer prevention, healthcare burden, patient acceptance, and effectiveness across different populations remain unsolved (Boege et al., 2024; Misawa et al., 2025).

Stewart *et al*., attempted to map ethico-legal principles for the use of AI in gastroenterology, while raising problems of participation, responsibility, and social license referring to statements of ethical principles for AI. These substantive principles included (i) respect for persons engagement, (ii) autonomy transparency, (iii) privacy and confidentiality supervision, (iv) beneficence accountability, (v) harm minimization, (vi) responsibility and (vi) justice/fairness. Procedural principles included (i) engagement, (ii) transparency and (iii) accountability/responsibility (Stewart et al., 2021).

## 5 Conclusion

AI has increasingly influenced gastroenterology by improving lesion detection, enhancing diagnostic accuracy, and supporting increasingly sophisticated clinical decision-making. Nevertheless, technological performance alone is insufficient to justify widespread clinical implementation. A “data tsunami” or “datanami” refers to the overwhelming, rapidly increasing volume of data generated in the modern digital age, often exceeding the ability of businesses and organizations to manage, process, or analyse it effectively. One of the most debated concepts of AI use in clinical research and practice is to delineate the thin and, sometimes, blur line that balances productivity and human value. Can we quantify (and therefore, give a percentage) on how much AI should be involved in treatment algorithm shifts and clinical decision making (Rojek et al., 2026)?

With progress come scepticism. From the initial application of AI in endoscopy limited to “edge detection” by identifying sharp changes in brightness, texture and “region growing” by a group of pixels of similar properties (useful for lesions with edges that were undetectable during standard endoscopy) medical devices are constantly evolving form generic to personalized solutions (Attardo et al., 2020). AI cost is challenging due to the lack of pre-existing data. Even though, the cost associated with new technologies tends to drop over time, cost estimation studies still need to be run and updated throughout the lifecycle of the AI application. However, this is not easily feasibly mainly due to the lack of real AI applications in clinical setting. To cut costs, the employment of existing or pre-trained models, the utilization of collaborative platforms and open-source tools, running models on existing hardware, the use of existing datasets the development of tools that allow for the automatization of routine and time-consuming tasks while prioritizing high-incidence conditions can provide logistic economic ease (Christou and Tsoulfas, 2023). An established modified Delphi approach for AI research priority setting only for colonoscopy some years ago, put forward several questions/themes that needed to be put into context (clinical trial design/end points, technological developments, clinical adoption/integration, data access/annotation and regulatory approval) (Ahmad et al., 2021).

In the field of gastroenterology, AI-assisted or driven diagnostic devices have produced clear cut improvements in early detection and accuracy while providing dynamic treatment adaptations and real-time monitoring in respect to treatment (Mohanadas et al., 2026). As clinical decision making has become increasingly complex and challenging for physicians, Large Language Models (LLMs) introduce new possibilities for Clinical Decision Support Systems (CDSS). The future looks even more promising with the inadvertent addition of -omics (genomics, transcriptomics, proteomics and metagenomics) in the field of AI research (Cannarozzi et al., 2024).

However, up to now, adoption is still limited as real-world performance and generalizability issues are still a struggle. For upper GI endoscopy, reviews report around 90–95% diagnostic accuracies for detecting early stage esophageal and gastric neoplasia when compared to endoscopies performed by non-experts. For lower GI endoscopy, CADe systems report a relative consistent 15–20% ADR increase while computer-assisted diagnosis (CADx) algorithms achieve a >90% accuracy in differentiating neoplastic from non-neoplastic polyps, supporting “resect-and-discard” strategies. Despite these benefits, barriers persist such as dataset bias, lack of generalizability, medicolegal ambiguity, and regulatory inconsistency; new exciting scientific processing of data can help bridge the gap by considering workflow integration, clinician training, alarm fatigue and regulatory issues (Yagnik et al., 2026).

Careful training and validation of AI tools seems to help bridge that gap. A cross-disciplinary example of an AI-generated assessment tool for sleep quality assessment (versus an established validated instrument such as the Pittsburgh Sleep Quality Index—PSQI) demonstrated only slight agreement, thereby suggesting that the AI tool could underestimate the sleep quality assessment compared to the PSQI scoring. Perhaps, reinforcing the importance of appropriate validation before translating AI performance into clinical practice can overcome this limitation (Dharani et al., 2025).

Domain adaptation, a tool that generates a newly converted image suitable for other domains, has shown promise in reducing the differences in appearance among the images collected from different devices. Domain adaptation is expected to improve the reading accuracy of AI for heterogeneous image distributions in GI endoscopy and medical image analyses (Wiest et al., 20250; Kim et al., 2023).

Future adoption will depend on demonstrating sustained improvements in patient outcomes, acceptable cost-effectiveness, equitable reimbursement strategies, transparent regulatory oversight, and responsible integration into routine clinical practice. Microsimulation modelling currently provides an important modelling approach linking improvements in diagnostic performance with long-term clinical and economic outcomes. However, continued prospective evaluation remains essential as AI technologies, healthcare systems, and reimbursement policies evolve. A microsimulation model schematic for AI-assisted endoscopy evaluation in colonoscopy, alongside main assumptions that must be met for this to be effective is visualized in Figure 3.

**Figure 3:**
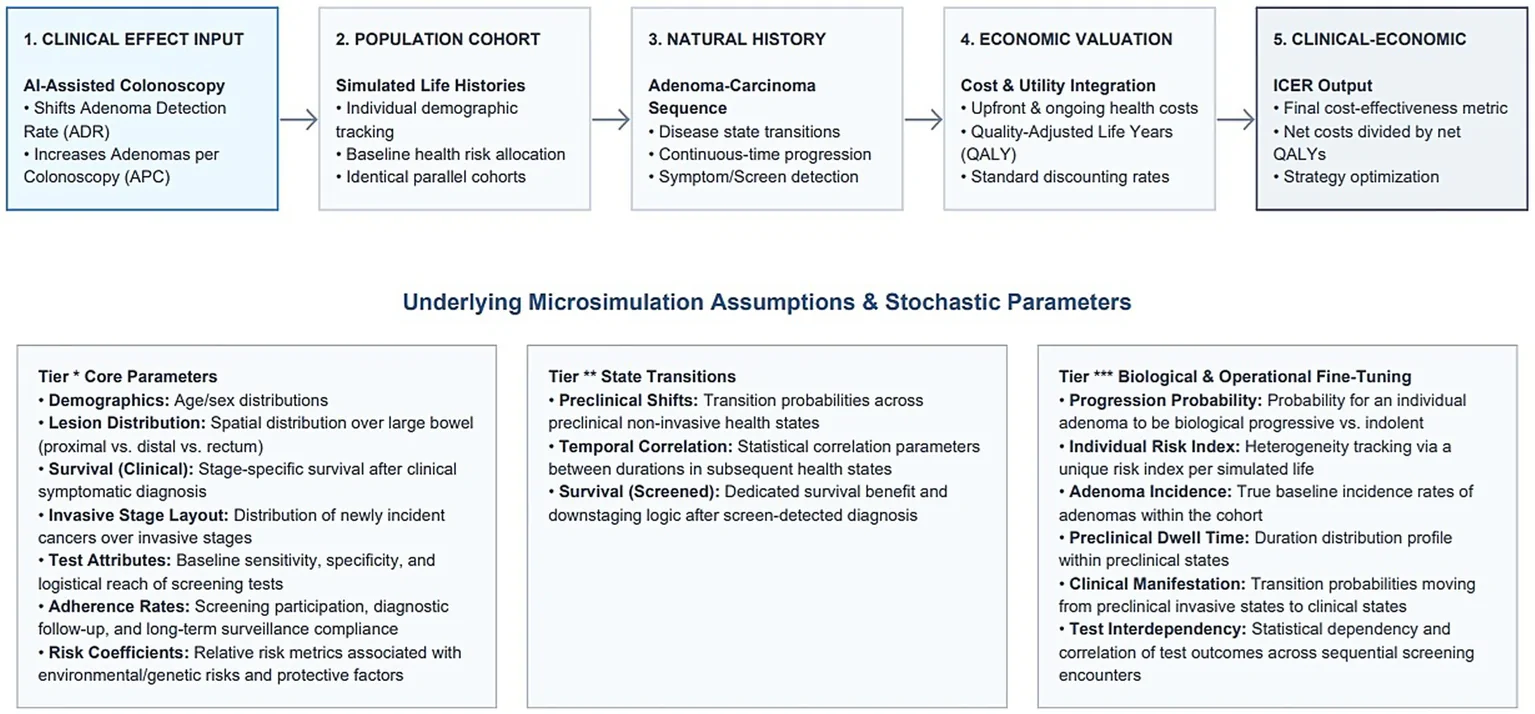
A microsimulation model schematic for AI-assisted endoscopy evaluation. A horizontal schematic mapping clinical efficacy parameter (ADR) through patient trajectories and natural history states to generate the Incremental Cost-Effectiveness Ratio (ICER).

Most importantly, the future of AI in gastroenterology should not be defined by replacing clinicians but by strengthening the partnership between computational intelligence and human expertise. Algorithms contribute speed, consistency, and analytical power; humans contribute clinical judgment, ethical reasoning, empathy, accountability, and wisdom. These capabilities are complementary rather than competing. The human-AI partnership section is of particular importance and could be strengthened by discussing calibrated reliance, preservation of independent clinical judgment, and the potential risk of deskilling. Yagnik et al., recently proposed human-in-the-loop safeguards, including independent assessment, AI-off training, disagreement review, and continued evaluation of the human-AI team (Yagnik et al., 2026).

Ultimately, the success of AI will be determined not by how intelligent the technology becomes, but by how effectively it improves patient care while preserving the human values that remain central to the practice of medicine. The future of AI in gastroenterology will not be determined by algorithms alone, but by the value they generate for patients, healthcare professionals, and society.
